# Properties and Bioapplications of Amphiphilic Janus Dendrimers: A Review

**DOI:** 10.3390/pharmaceutics15020589

**Published:** 2023-02-09

**Authors:** Adina Căta, Ioana Maria Carmen Ienașcu, Mariana Nela Ştefănuț, Dan Roșu, Oana-Raluca Pop

**Affiliations:** 1 National Institute of Research and Development for Electrochemistry and Condensed Matter, 144 Dr. A. P. Podeanu, 300569 Timişoara, Romania; 2 Department of Pharmaceutical Sciences, Faculty of Pharmacy, “Vasile Goldiș” Western University of Arad, 86 Liviu Rebreanu, 310045 Arad, Romania; 3 Faculty of Pharmacy, University of Medicine and Pharmacy “Victor Babeș” Timișoara, 2 Eftimie Murgu Square, 300041 Timișoara, Romania

**Keywords:** dendrimers, amphiphilic Janus dendrimers, biomedical applications, dendrimersomes, glycodendrimersomes

## Abstract

Amphiphilic Janus dendrimers are arrangements containing both hydrophilic and hydrophobic units, capable of forming ordered aggregates by intermolecular noncovalent interactions between the dendrimer units. Compared to conventional dendrimers, these molecular self-assemblies possess particular and effective attributes i.e., the presence of different terminal groups, essential to design new elaborated materials. The present review will focus on the pharmaceutical and biomedical application of amphiphilic Janus dendrimers. Important information for the development of novel optimized pharmaceutical formulations, such as structural classification, synthetic pathways, properties and applications, will offer the complete characterization of this type of Janus dendrimers. This work will constitute an up-to-date background for dendrimer specialists involved in designing amphiphilic Janus dendrimer-based nanomaterials for future innovations in this promising field.

## 1. Introduction

The dendritic (or tree-like) configuration is one of the most widespread patterns observed in nature, both in the non-living world (e.g., lightning, snowflakes, river networks, mountains) and biological systems (e.g., tree branching/roots, flowers, lungs and vasculatory system, neuronal system, bacterial colonies, stony corals) [1,2,3]. These natural tree-like patterns have been a real source of inspiration for chemists who tried to reproduce the well-defined branched structure from nano to macromolecular levels.

Although the concept of highly branched structures in macromolecular systems was initially proposed by Flory in the early 1940s [4,5,6], the first example of branched macromolecules obtained by “cascade syntheses” was reported in 1978 by Vögtle’s group [7]. The first article using the term “dendrimer” was published in 1985 by the Tomalia group [8] describing in detail the synthesis of polyamidoamine dendrimers (PAMAM) by “time sequenced propagation techniques”. Independently, in the same year, Newkome et al. [9] reported the synthesis of “monocascade spheres (arborols)”.

The term “dendrimer” originates from the Greek words ‘*Dendron*’, meaning “tree”, and ‘*meros*’, meaning “part”. Dendrimers are three-dimensional macromolecular entities with radial symmetry having a globular, tree-like structure and a large number of functional groups [10].

Typically, they are composed of three main structural parts: a central core, repeating units, and surface terminal groups [11,12]. The repeating branching units of dendrimers are organized around a focal point, and on the outside, they present an increasing number of exposed terminal groups that can be functionalized, thereby changing their physicochemical or biological properties [13,14].

Due to their unique structures and characteristics, dendrimers are suitable for a wide variety of applications in various fields such as: biomedicine [15,16,17,18], drug delivery [19,20,21,22,23], tissue engineering [24,25], catalysis [26,27,28,29], sensing [30,31,32,33], imaging [34,35,36], hybrid materials [37,38], and solar cells [39,40,41,42].

These compounds have sparked a large number of publications and many expectations, especially in the medical field, but their application in clinical studies has been very weak so far [43]. However, a very recent example refers to a dendrimer (hydroxyl-polyamidoamine dendrimer–*N*-acetylcysteine conjugate) therapy against severe COVID-19 that has entered into Phase II clinical trial and proved the attenuation of inflammatory and neurological injury markers [44]. In spite of their advantageous characteristics and the great potential as drug delivery carriers, dendrimers present also certain limitations such as: difficulties associated with purification [45], toxicity in biological systems, especially of cationic dendrimers [16,46,47,48], difficulties in obtaining consistent drug loading efficiencies and controlled drug release [49], and non-degradability in the physiological environment, causing side effects induced by their accumulation in cells and tissues [50].

To counteract the limitations of symmetrical conventional dendrimers, several strategies have been proposed and developed by different scientists. For example, in recent years, many researchers have focused on the development of biocompatible and biodegradable dendrimers [51,52,53]. The use of biodegradable dendrimers allows the excretion or elimination of non-toxic small dendritic fragments through metabolic pathways [50,54]. A common way to improve the biocompatibility and bioactivity of conventional dendrimers and to reduce their toxicity is chemical modification of their surface, the peripheral charge modifications having a key influence for enhancing their biocompatibility and precisely customizing their properties [55,56,57,58,59].

Furthermore, dendrimers can also be designed to incorporate regions of chemically and structurally distinct groups [49]. These dendrimers are known as “Janus dendrimers” (JDs), named after the ancient Roman god “Janus”, usually depicted as having two faces. In recent years, this new class of dendrimers also known as diblock dendrimers, ‘‘surface-block’’ dendrimers, block co-dendrimers, diblock co-dendrimers, asymmetrical or bow-tie dendrimers [60], aroused the interest of researchers due to their asymmetric structures and their potential to overcome some of the limitations of conventional dendrimers.

In 1989, Casagrande et al. [61] reported the first “Janus beads”, glass spherical particles with diameters in the range of 50–90 µm with one hemisphere hydrophilic, and the other side hydrophobic. They studied the properties of these unique amphiphilic solids at oil/water interfaces and noted their special behavior and the promising advantages for further development and applications. This study triggered the interest of the Nobel Laureate, Pierre-Gilles de Gennes. In 1991, in his Nobel Prize lecture entitled “Soft matter”, he presented “Janus grains”, particles having two sides, one polar and one non-polar [62]. Since then, these asymmetric structures have fascinated the scientific community and research in the field has expanded far beyond the initial two-faced amphiphilic structure towards more complex structures, including Janus dendrimers.

**Figure 1:**
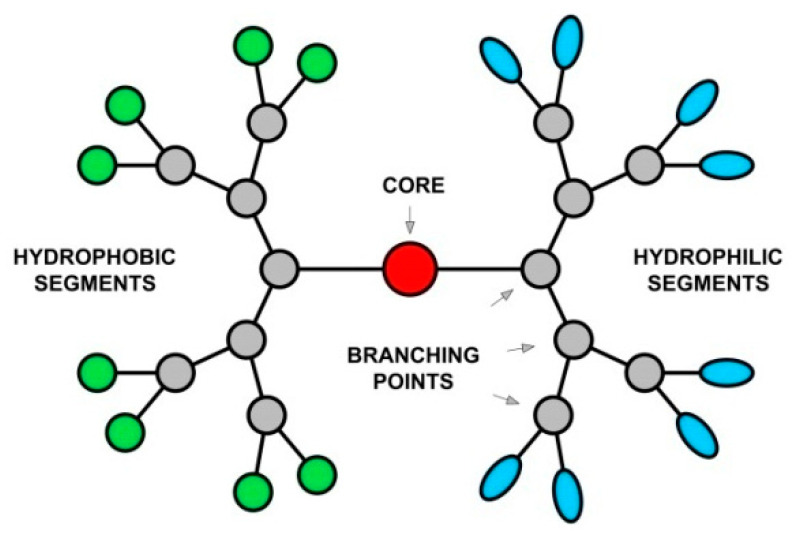
Schematic representation of an amphiphilic Janus dendrimer.

**Figure 2:**
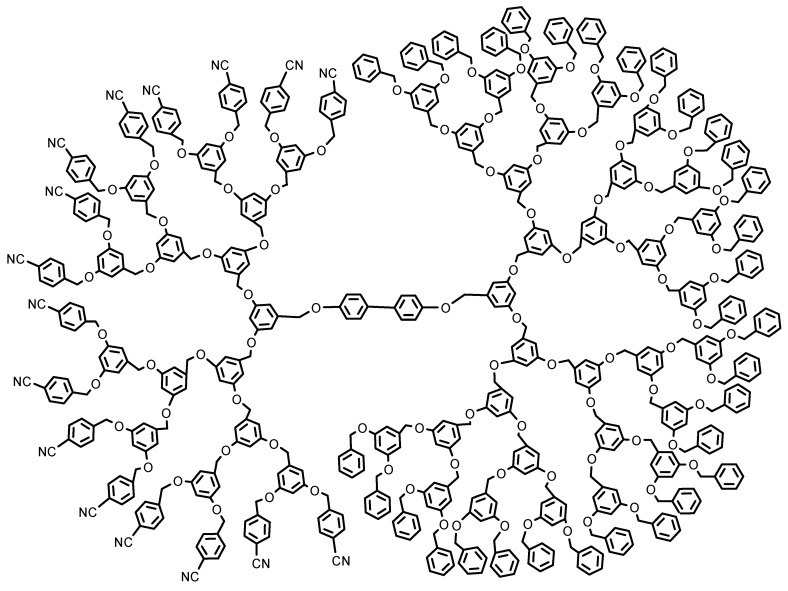
A dipolar dendritic molecule designed by Fréchet group [65] The broken symmetry structure of Janus dendrimers and their self-assembly ability to form various complex molecular architectures give them the flexibility and versatility to be applied in different fields such as biomedicine (especially as nanocarriers) [63], molecular imaging, especially MRI (magnetic resonance imaging) [74,75,76,77], optoelectronics [78,79,80,81,82], catalysis [83], ionic liquids [84,85], and thermal actuators [86].

The general structure of Janus dendrimers consists of two dendrimeric halves with different terminal functions joined through the core [60]. They are synthesized by coupling two different dendrons in terms of size and functionality to obtain a single amphiphilic or heterofunctional molecule with distinctive properties [63]. A schematic representation of an amphiphilic Janus dendrimer is illustrated in Figure 1. The synthesis of unsymmetrically surface-functionalized dendritic molecules (Figure 2) was initiated by Fréchet and co-workers in the early 1990s [64,65], then in the following years, the research in this field has expanded. In recent years, a crucial contribution in the development and investigation of Janus dendrimers was made by the research group of the American of Romanian origins chemist Virgil Percec. They developed new synthetic routes leading to several distinct libraries of uncharged or positively charged amphiphilic Janus dendrimers, and studied their supramolecular assembly in water into giant dendrimersomes with excellent mechanical properties [66]. In addition, they reported the first attempt to predict the dimensions and some properties of monodisperse dendrimersomes and demonstrated that the molecular structure of amphiphilic Janus dendrimers determines the morphology of the supramolecular assemblies [67]. They synthesized the first examples of amphiphilic Janus glycodendrimers (sugar-containing dendrimers) that can self-assemble in aqueous solutions, leading to monodisperse vesicles named glycodendrimersomes (GDSs) and studied their interactions with lectins [68,69,70,71]. Additionally, the designing of a one-component multifunctional sequence-defined ionizable amphiphilic Janus dendrimer (IAJD) delivery system for mRNA [72,73] could have a profound impact on the future of genetic nanomedicine.

The present review will focus on the pharmaceutical and biomedical application of amphiphilic Janus dendrimers. Important information for the development of novel optimized pharmaceutical formulations, like structural classification, synthesis, properties and applications, will offer the complete characterization of this type of Janus dendrimers. This work insists on the newest published papers in Janus dendrimers area, so dendrimer specialists concerned in design and applications of Janus dendrimers will fully benefit from the latest knowledges in the field.

## 2. Definition and Nomenclature of Amphiphilic JDs

Amphiphilic Janus dendrimers (JDs) can be defined as dendritic macromolecules made of two dendrons with opposite polarities, which either differ from each other by their terminal groups, or their structures differ entirely [87]. Amphiphilic JDs are named following a more or less general scheme that encompass in this order, the lipophilic dendrons, the core if exists, and the hydrophilic dendrons. Sherman and co-workers brought a remarkable contribution in this regard, modifying the specificity-lacking nomenclature of reviewed JDs [88].

## 3. Classification of Amphiphilic JDs

Among JDs, two significant categories are distinguished, JDs: amphiphilic Janus dendrimers and amphiphilic Janus glycodendrimers.

Amphiphilic Janus dendrimers can be divided into three groups, twin−twin, with two lipophilic dendrons coupled to two hydrophilic dendrons, twin−single, with two lipophilic dendrons coupled to one hydrophilic dendron, and single−single, with one lipophilic dendron coupled to one hydrophilic dendron. Depending on the presence of specific moieties in their molecule or based on their special architectures, amphiphilic Janus dendrimers can be divided into fluorinated JDs, Janus metallodendrimers, hyperbranched JDs, and so on [88].

Amphiphilic Janus glycodendrimers (JGDs), containing sugars like D-galactose, D-mannose, and D-lactose in their structure, can be classified into twin−twin, with two lipophilic dendrons linked to two glycosylated hydrophilic dendrons, single−single, containing one lipophilic dendron linked to a glycosylated hydrophilic dendron, and twin−mixed, with twin hydrophobic dendrons linked to one hydrophilic and one glycosylated hydrophilic dendron. If the sugar sequence and density are well-defined, JGDs are called sequence-defined JGDs [88].

**Figure 3:**
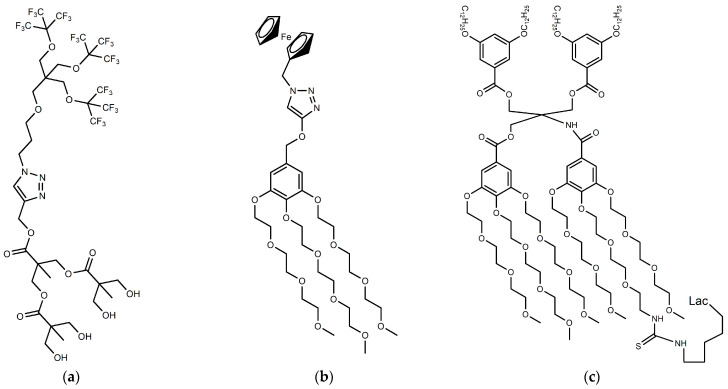
Examples of particular Janus dendrimers (**a**) fluorinated JDs [77]; (**b**) Janus metallodendrimers [93]; (**c**) sequence-defined JGDs [92].

Some examples of particular JDs, recently designed, are shown in Figure 3.

Percec and co-workers reported extensive libraries of twin–twin [66,68], single–single [89] and sequence-defined [70,90,91,92] amphiphilic Janus dendrimers and glycodendrimers.

## 4. Characteristics of Amphiphilic JDs and Dendrimersomes (DSs)

According to their definition, amphiphilic JDs benefit from the characteristics of dendritic macromolecules, like defined structure and opportunity of expanded functionalization, and from the features derived from their amphiphilic character, such as the possibility of self-assembling in different media [87]. Considering the unique structure of amphiphilic JDs, which results from the controllable size, assembly, density, generation and number of terminal groups of their dendrons [87], and the enhanced solubility, along with the presence of internal cavities, the possibility of using JDs in various applications can be clearly predicted [94].

Amphiphilic JDs are molecules comprising both of polar and non-polar dendrons, which is the main reason for their self-assembly in water forming complex molecular architectures. These form stable structures with uniform size and various chemical functionalities [94].

The injection, thin-film hydration, and oil-in-water methods were used for the self-assembly of JDs to form uniform dendrimersomes (DSs) and other varied complex architectures including tubular vesicles, cubosomes, and micellar structures such as rods, disks, and helical ribbons [66,68,86,88].

Generally, DSs showed predictable thickness and diameter and outstanding stability in time and many environments, even in the presence of competitive guest or host molecules [88]. There were reported DSs stable in the first 50 days after preparation at 25 °C in ultrapure water, and sometimes with a constant size for up to 244 days at room temperature, or DSs stable upon annealing from 22 to 80 °C, with almost a constant size, but also DSs which displayed an evident increase in size when heated to at least 50 °C [66]. Some DSs also demonstrated the ability to retain most of their cargo under physiological temperature and pH 7.4 for over 100 h [66]. DSs self-assembled from the hyperbranched JDs were stable in size and morphology after 6 months at room temperature, or when heated from 20 to 70 °C [95]. Good stability after photopolymerization and in buffer solutions was reported in the case of some DSs, even when more JD was added [96]. To enhance the stability of DSs, the co-assembly stabilization method using an anionic lipid that can reduce electrostatic forces was also reported. Such stabilized DSs unveiled payload retention and stability comparable to that of conventional liposomes [74]. Obviously, there are also examples of DSs with poor retention and deficient stability in human serum at physiological temperature [74].

Along with stability, many DSs proved to have also minimal toxicity both in vitro and in vivo [88]. Some DSs exhibited levels of toxicity similar to polymersomes, in some cases with no significant reduction in cell viability [66]; their breakdown products, i.e., phenolic acids, are known to lack toxicity [88]. On the other hand, great biocompatibility was obtained for DSs containing siRNA [97,98], the evaluation of inflammatory cytokines revealing no substantial inflammatory response [97]. More examples of DSs stability and toxicity are presented in Section 7.

## 5. Synthesis of JDs

Conventional dendrimers have been mainly synthesized by two major approaches, the divergent and the convergent growth approach, respectively. In the divergent approach [8,9], the synthesis of the dendrimer starts from a multifunctional core by adding building blocks step-by-step to the periphery. The convergent method, first reported by Hawker and Fréchet [99], involves the construction of dendrimers from the periphery to the core, by preparing initially branched dendrons (dendrimer wedges), which are finally attached to the core [14,100]. In addition, different accelerated growth methods (the third approach) that combine the convergent and divergent strategies, such as multigenerational coupling (hypermonomers, hypercores, double exponential growth), and orthogonal and chemoselective syntheses have been developed [13,15,100,101].

The Janus dendrimers era started with the work of Fréchet and co-workers (1991) [65] who obtained the first example of all types of Janus dendrimers by controlled grafting of a dendron at a multifunctional core level, followed by the grafting of the second dendron to the unused function of the core (convergent approach). However, the first example that recognized and used the term Janus dendrons/dendrimers was published by Tomalia group in 2006 (PCT-WO 2006/195043 A2) and subsequently patented in 2011 [102]. Their invention refers to the specific synthesis of new Janus dendrimers where at least two dissimilar dendrons having a heterobifunctional character are joined at their cores. These Tomalia-type Janus dendrimers, as well as many others, have been extensively reviewed by Tomalia et al. [103].

Until 2020, Janus dendrimers were synthesized via the three general approaches employed for the obtaining of classical dendrimers [60,63], all of them based on designing the dendrons through convergent [104,105] or divergent processes [106,107,108].

**Figure 4:**
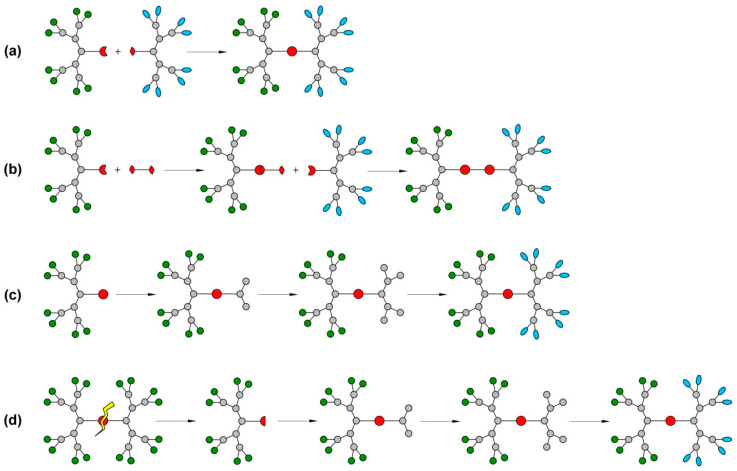
Synthesis of amphiphilic Janus dendrimers (**a**) chemoselective coupling; (**b**) heterogeneous double exponential growth; (**c**) mixed modular approach; (**d**) secondary growth of the dendron from the broken structure of a conventional dendrimer.

Currently, Janus dendrimers can be synthesized by four major strategies (Figure 4), including the chemoselective coupling approach where dendrons with complementary functions are coupled together [106,107,108], the heterogeneous double exponential growth approach in which the dendrons are attached one at a time to a multifunctional core [104,105], the mixed modular approach where a focal point of a dendron is used for the growth of new branches [109,110], and the secondary growth of the dendron from the broken structure of a conventional dendrimer approach [94,111], an original method proposed by Najafi et al. (2020). This novel fourth approach consists of the synthesis of a classic symmetrical dendrimer, followed by the splitting of its structure with obtaining a dendron which will subsequently undergo the third approach in order to yield JDs.

## 6. Characterization Methods

Janus dendrimers are asymmetric homogeneous branched architectures with two dendrimer wedges (dendrons) linked at a functional core. Their structural diversity relies on the variety of the repeating units of the dendrons and terminal groups attached on their surface. Because of their different structures and applications, the characterization of JDs represents a challenge, especially when only well-defined structures are suitable for obtaining safe and effective drug carriers.

Different analytical techniques, concerning both molecular chemistry to attest the step-by-step synthesis accomplishment and polymer chemistry to confirm their repetitive structure of monomers, have been employed for JDs characterization. Spectroscopic and spectrometric methods like infrared spectroscopy (IR), UV-vis spectroscopy (UV-Vis), fluorescence spectroscopy, nuclear magnetic resonance (NMR), X-ray diffraction (XRD) and mass spectrometry (MS), are widely used to confirm the structure and identity of JDs. The presence of some terminal groups was demonstrated using electrochemical methods such as cyclic voltammetry (CV). To study the morphology, size and shape of JDs, microscopic techniques such as scanning electron microscopy (SEM), transmission electron microscopy (TEM), and atomic force microscopy (AFM) were employed. Dynamic light scattering (DLS) is used to estimate some properties of JDs in solution; more precisely, the diffusion coefficient can be measured and used to determine the hydrodynamic diameter of particles. Size exclusion chromatography (SEC) allows the determination of the purity and monodispersity of JDs. Along with structural defects and purity of JDs, the characterization methods also proved to be helpful in predicting possible in vivo interactions between JDs and biological entities. Some examples of the analytical methods used for characterization of JDs are presented in Table 1.

Obviously, there are more analytical techniques used by researchers to characterize the behavior of JDs. For instance, confocal laser scanning microscopy (CLSM) was employed in the study of vesicle dispersion, and probed the internal onion-like structure of some glycodendrimersomes assembled from JDs [112]. The capacity of cholesterol-based JDs to self-assemble was evaluated using a steady-state fluorescence technique with N-Phenyl-1-naphthylamine (NPN) as fluorescence molecule [113]. X-Ray diffraction analysis showed the network of hydrogen bonds between hydroxyl groups and triazole rings of neighbor molecules of fluorinated JDs [77]. X-Ray scattering (SWAXS), polarized optical microscopy (POM), and differential scanning calorimetry (DSC) were also used to study the molecular organization of fluorinated JDs in bulk. These analytical methods showed the loss of crystallinity when the dendritic generation increases, and cohabitation of diverse polymorphs with a various thermal behavior in case of FJD3 [77].

**Table 1:** Examples of analytical methods used for characterization of JDs.

Hydrophilic/Hydrophobic Dendrons	Methods	Tests	Results	Conclusions
TEG termini /Ferrocenyl (Fc) units [104]	^1^H-NMR	Structure elucidation	- absence of CH_3_O- protons signal - presence of CH_2_ protons adjacent to the ester group	- success of the esterification reaction between the two dendrons
	MS	Molecular mass	- molecular peak - no molecular peak, fragment peaks	- in good agreement with the theoretical value - large molecular weight and easy C=O bond cleavage
	UV-Vis	Structure elucidation	- λ_max_	- d-d* transition of Fc groups
	CV	Electrochemical properties Structure elucidation	- single oxidation wave for all Fc groups - no adsorption in the CV	- total no. of e^−^ transferred in the oxidation wave is equal to the no. of Fc units
	AFM	Self-assembly capability	- images of amplitude profiles	- particle with approx. spheroidal morphologies - average particle diameter
	DLS	Self-assembly capability	- hydrodynamic diameter	- polydispersity index
TEG termini /Ferrocenyl (Fc) units [93]	^1^H-NMR	Structure elucidation	- presence of triazolyl H^+^ signal - presence of free/substituted Cp H^+^ signal	- success of the click reaction - integrity of the Fc group
	MS	Molecular mass	- molecular peak	- in good agreement with the theoretical value
	UV-Vis	Structure elucidation	- λ_max_	- d-d* transition of Fc groups
(NH_3_^+^)_8_ [GMPA]/ [MPA](C17)_4_ [114]	^1^H-NMR	Structure elucidation	- presence of signal for the triazolyl proton	- success of the click reaction - integrity of the Fc group
	MS	Molecular mass	- [M-Na]^+^ molecular peak	- in good agreement with theoretical value
	SEC	Structure elucidation	- single and symmetrical monomodal peak	- monodispersity of JDs - absence of unreacted dendrons
	TEM	Morphology of JD aggregates	- rounded micelles, homogenous size distribution	- well-defined Janus structure
	DLS	Morphology of JD aggregates	- hydrodynamic diameter	- different values for designed JDs
bis-MPA (G1-G3)/F_27_-N_3_ [77]	^1^H-NMR	Structure elucidation	- presence of signal for the triazolyl proton	- success of the click reaction
	^19^F-NMR	Structure elucidation	- presence of one single peak	- presence of only one fluorinated pure compound
	FTIR	Structure elucidation	- the disappearance of the N=N=N azide stretching bands and C−H stretching bands	- success of the click reaction
	HR-MS (ESI)	Molecular mass	- [M-Na]^+^ molecular peak	- in good agreement with the theoretical value
	SWAXS	Molecular organization of JDs in bulk	- cubic mesophase which freezes in a glassy solid state on cooling to r.t.	- high tendency of fluorinated moieties to microsegregate - coexistence of different polymorphs with different thermal behavior (JDG3)
	POM	Molecular organization of JDs in bulk	- total absence of birefringence for JDG2	- the formation of a cubic mesophase (JDG2) - coexistence of different polymorphs with different thermal behavior (JDG3)
	TGA	Molecular organization of JDs in bulk	- JDG1,2 show a single-step degradation - JDG3 shows a two-step degradation	- good thermal stability of all FJDs - thermal degradation for JDG3 starts from the outermost layer and then extends to the inner layers
	DSC	Molecular organization of JDs in bulk	- sharp/broad melting point peaks	- loss of crystallinity upon increasing the dendritic generation - coexistence of different polymorphs with different thermal behavior (JDG3)
	Cryo-TEM	Morphology of JD assemblies	- micelles/larger spherical NPs (JDG1-2) - well-defined DSs: vesicles, sheets, helices, tubules	- coexistence of two families of nanoparticles (JDG1-2) - peculiar self-assembly behavior upon aging (JDG3)
	DLS	Morphology of JD assemblies	- hydrodynamic diameter - polydispersity and hydrodynamic radius increased significantly upon aging (JDG3)	- no significant changes in size and polydispersity during sample aging (JDG1-2) - change in the aggregate morphology (JDG3)
	^19^F-NMR	Structure elucidation Morphology of JD assemblies	- presence of one single peak - strong quenching of the ^19^F NMR signal (JDG3)	- presence of only one fluorinated pure compound - reduced mobility of CF_3_ groups (JDG3)

Some of the methods presented above are also used for the characterization of existing interactions between JDs and drugs, and to certify the success of loading when the latter are encapsulated in such dendrimers. In case of using JDs as nanocarriers, other methods for studying the drug wettability and stabilization, the nature of physical interactions and the chemical compatibility of the mixtures were employed, including contact angle tensiometry (CAT), isothermal titration calorimetry (ITC), differential scanning calorimetry (DSC), and surface plasmon resonance (SPR). Table 2 presents the methods implemented for the characterization of some drug-JDs aggregates.

## 7. Applications of Amphiphilic JDs

**Figure 5:**
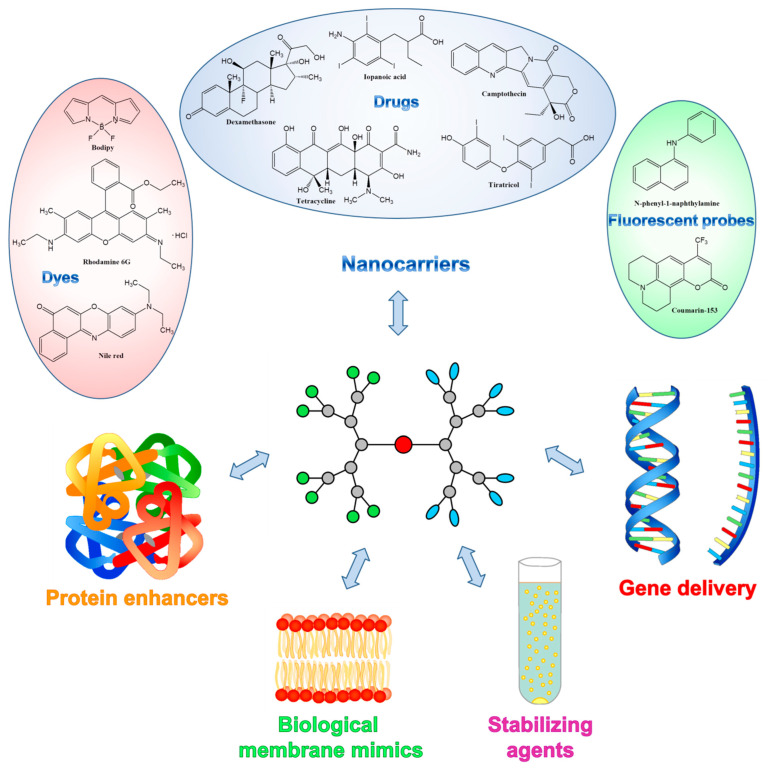
Biomedical applications of amphiphilic Janus dendrimers.

The unique architectures of amphiphilic Janus dendrimers, with multifunctional terminal groups, different structures of branches in a single molecule (beside the capacity of self-assembly in aqueous media forming dendrimersomes, which in turn benefit from properties like predictable size and thickness, stability and biocompatibility), constitute premises for a wide range of biomedical applications (Figure 5) where conventional dendrimers have failed [87,94].

### 7.1. JDs as Stabilizing Agents

#### 7.1.1. Drug Suspensions

The formulation of pharmaceutical nanosuspensions requires the presence of stabilizers, which act via electrostatic or steric interactions and enhance the dissolution of poorly water-soluble drug molecules. Amphiphilic Janus dendrimers can be adsorbed onto drug particles and provide steric stabilization against aggregation and recrystallization, offering a versatile platform for general use as stabilizing agents for drug suspensions [106].

**Figure 6:**
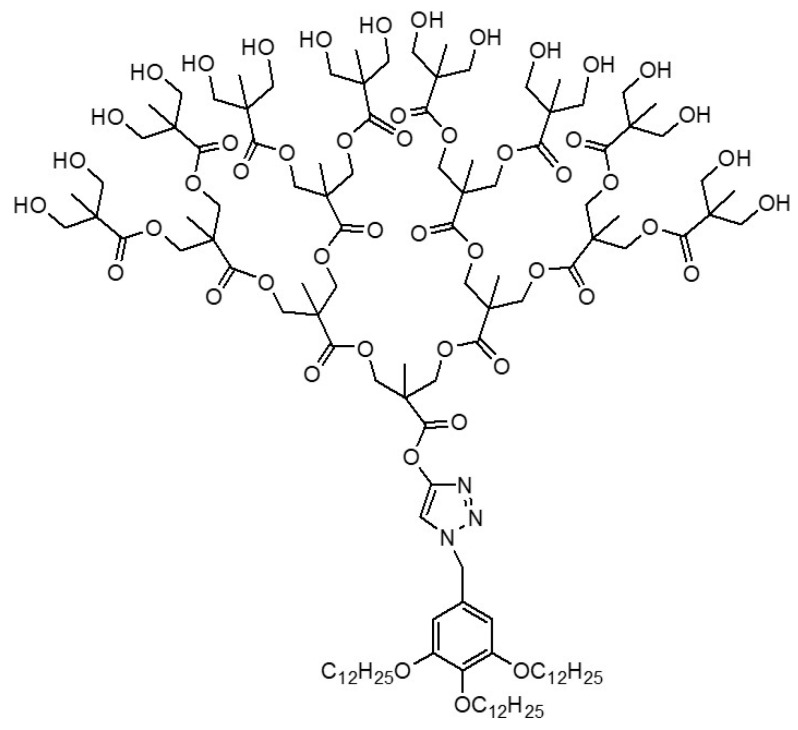
G4 JDs as stabilizers in media milling and dry-state processing of pharmaceuticals [106].

Thus, four new amphiphilic Janus dendrimers with hydroxyl-terminated bis-MPA dendrons and dodecyloxy chains were obtained and tested for their ability to stabilize colloidal drug suspensions through steric stabilization (Table 2). JDs adsorb onto indomethacine surfaces, forming hydrophobic interactions, and the adsorption kinetics are strongly related to the number of hydrophobic alkyl chains. These experiments proved the potential of such G4 JDs as stabilizers in nanomilling (Figure 6) and other steps of dry-state processing of drugs [106].

#### 7.1.2. Au and Ag Nanoparticles

Silver and gold NPs are very important for nanoscience and nanotechnology due to their numerous applications in catalysis, materials science, optical biosensors, and nanomedicine [93,115,116].

Janus metallodendrimers incorporate metal-containing moieties into the structure of JDs and benefit from the exceptional combination of organic and inorganic components, with outstanding catalytic, electronic, magnetic and radioactive properties [93].

Thus, ferrocene (Fc)-containing JDs can be used as neutral single-electron transfer agents for the reduction of Au(III) and Ag(I) in the synthesis of AuNPs [117,118,119,120,121,122,123,124,125] and AgNPs [123,124,125,126,127,128].

Few experiments regarding the preparation of silver and gold NPs using water-soluble Fc-containing dendrimers as both, stabilizers and reductants, in water systems involving no external reductant are available [93,104]. The use of external reducing agents, such as NaBH_4_, along with Fc-containing dendrimers for obtaining AgNPs and AuNPs, are highlighted in some works [93,104,129].

Hence, two novel amphiphilic triazolylferrocenyl with TEG termini JD (Table 1) were obtained by the click reaction between azidomethylferrocene and TEG-terminated acetylenes, and tested as neutral ligands for the AgNPs-1 and AuNPs-1 stabilization in water in the presence of NaBH_4_ as a reducing agent for Ag(I) and Au(III). In the reaction between JDs and Ag(I) or Au(III), the significant impact of the electronic properties of the ferrocenyl-core linker on the viability of the redox reaction, and the ability of this kind of JDs to manage such redox processes was proven. The authors also highlighted the potential of ferrocenyl-containing JDs with varied types of redox active units for extending the spectrum of properties in this field [104].

Redox-responsive Fc-containing polymers could also be used as drug carriers, ensuring manageable processes of delivery and release of the drugs due to the reversible conversion between hydrophobic Fc and hydrophilic ferrocenium groups via chemical and electrochemical reduction, offering them the capacity to form reversible self-assembly systems [93,130,131].

### 7.2. JDs as Biological Membrane Mimics

The understanding of various cellular processes needs some artifacts like biological reconstitution of the cell membranes, thus a significant interest in designing synthetic analogs and models of the membrane components is granted.

Two important components of cell membranes are phospholipids and glycolipids. Phospholipids can be mimicked by amphiphilic JDs which have the ability to self-assemble in different liquid media into stable dendrimersomes (DSs) [88,132,133] with membrane thickness similar to phospholipids [88]. Janus glycodendrimers (JGDs) obtained by conjugation of sugars with JDs, were able to self-assemble in buffer solutions [71,88,91,134] into glycodendrimersomes (GDSs) which mimic glycolipids.

Therefore, dendrimersomes and glycodendrimersomes could successfully substitute liposomes if we consider their special features like tunable size [67], structural organization [69,71], enhanced stability [69,133], biocompatibility, similar membrane to living cells [88] and functional surfaces [71].

Based on the studies on the coassembly into hybrid vesicles of block copolymers, natural phospholipids and membrane proteins [135,136,137,138,139], DSs and GDSs have also been joined with phospholipids, membrane proteins, bacterial cell membranes, etc., forming hybrid architectures with unique morphologies that lend themselves to interesting biological applications.

#### 7.2.1. Amphiphilic Janus Dendrimers (JDs)

In order to stabilize the cell membranes and vesicles that have originated from human cells, Yadavalli and coworkers (2019) used DSs obtained from appropriate JDs containing hydrophobic 3,5-di-dodecyloxybenzoic ester dendrons and hydrophilic 3,4,5-tris-triethylene glycol benzoic ester dendrons. They also proved the value of such DSs as potential cell-targeting agents by combining them with bacterial membrane vesicles holding a bacterial adhesin protein (YadA), generating a cell-like hybrid vesicles recognized by the living human cells. Using DSs, the development of synthetic and hybrid protocell capsules to perform cell-like functions such as recognition, signaling, and delivery, seems tangible [133].

Natural polyphenols and phenolic acids possessing dodecyloxy chains were employed as building blocks for designing new amphiphilic JDs as cell membrane mimics, with bilayer thickness comparable to the natural phospholipids having 16 or 18 carbons in the alkyl groups. Unilamellar DSs were obtained in higher yields and shorter reaction times via self-assembly of such programmable JDs, and have the potential to act as significant tools for synthetic cell biology, encapsulation, and delivery [140].

A new type of zwitterionic JD, consisting of a zwitterionic phosphocholine hydrophilic headgroup and a 3,5-substituted dihydroxybenzoate-based hydrophobic dendron, was introduced by Joseph et al. [141]. This type of JDs self-assembles in water into zwitterionic dendrimersomes (z-DSs) that closely mimic the thickness, lateral mobility, and flexibility of natural cell membranes.

The combination of the two key features of cell-like DSs assembled from JDs, i.e., high flexibility and stability, confers them the ability to engulf bacteria. Kostina et al. (2019) synthesized new JDs which self-assemble into unilamellar vesicles with a biomimetic thickness, high flexibility and stability conferred by dodecane hydrophobic chains, and ultra-low adhesion to bacteria owed to the tri(ethylene oxide) hydrophilicity. These cell-like DSs proved superior to natural liposomes and synthetic polymersomes in terms of living bacteria endocytosis, emphasizing the opportunities of using the dendrimersomes in biomedical field [142].

Kostina et al. (2021) studied the influence of variation of amphiphile topology in the bilayer of some photoactive/stable JDs on shape changes of such cell membrane models, and demonstrated the utility of DSs to simulate and explain some cellular processes occurring in living cells [143].

Torre et al., 2019 considered the rebuilding of synthetic cells starting from compartmentation, encapsulation, and surface ornamentation of unilamellar and onion-like DSs self-assembled from amphiphilic JDs. They propose an approach for the modularly binding of proteins, nucleic acids, and hydrophobic target compounds to the edge of the DSs, demonstrating their value as adaptable synthetic biological membranes [132].

#### 7.2.2. Janus glycodendrimers (JGDs)

Janus glycodendrimers (JGDs), synthetic macromolecules mimicking glycolipids, were proved to self-assemble into nanovesicles presenting carbohydrates on their external surface, like the glycocalyx layer of eukaryotic cells, bacteria, and viruses.

Glycodendrimersomes (GDSs) are feasible mimics of biological membranes, i.e., glycans, that can serve to explain the structure and function of glycans. GDSs are self-assembled nanovesicles with cell surface-like programmable structures comprising carbohydrates moieties, which can be combined with lectins to discover the complexity of glycan-lectin specificity. Clinically, GDs could be used to target the nanoparticles to lectins in vivo and to specifically scavenge a lectin at sites of damaging action [71]. Xiao and coworkers (2018) synthesized Janus glycodendrimers with various sugar headgroups and coupled them with homodimeric, heterodimeric, and chimera galectins, to disclose the degree of cross-linking of the biomimetic nanoscale vesicles having both charged and uncharged ligands, hence contributing to elucidate the nature of these vital interactions [71].

Other exceptional Janus amphiphilic glycopeptide dendrimers for the biomimicry of glycans exhibited strong and specific recognitions with C-type mannose-specific lectin [144].

Rodriguez-Emmenegger (2019) obtained GDs acting as cell-membrane mimics, with hierarchical morphologies resembling bicomponent rafts starting from JGDs containing Lac and triethylene glycol. Their structures encode biological recognition and permit to reduce sugar–sugar interactions, allowing stronger binding to proteins, which recommend them for applications in cellular biology and nanomedicine [91].

Some JGDs were obtained by an isothiocyanate–amine coupling reaction among isothiocyanate-containing sequence-defined JDs, and linear or branched carbohydrates containing oligosaccharide and hydrophobic amino-pentyl units. These structures containing thiourea groups, which enhances the hydrophilic character of the *N*-pentyl linker, self-assemble into nanovesicles with lamellar surface, that mimic the recognition assemblies of the cell-surface of glycans and viral glycoproteins and displayed the potential of elucidating the ability of viruses to camouflage in order to avoid recognition [92].

Similar results, regarding the important influence of the thiourea interconnecting group placed amid the hydrophobic and hydrophilic dendrons of JDSs to their self-assembly, were obtained by Murphy et al. (2021). They also proved the stabilization of glycolipid-rich rafts and association of sulfatide-rich regions with specific glycoproteins [134].

Various pathogenic bacterial strains act by infecting the host cells through multivalent host-guest connections [145]. In order to find optimized carbohydrates-containing dendrimers nanostructures known for their ability to capture bacterial strains [69], Krishnan et al. (2020) designed carbohydrate-based Janus dendrimers that spontaneously self-assemble into high aspect ratio 2D sheets. The conjugation between the hydrophilic dendron containing galactose and hydrophobic tetraphenylethylene (TPE) dendron formed two JDs that proved to be multivalent ligands for bacteria able to capture and agglutinate them and to inhibit the bacterial proliferation [146].

Kostina et al. (2021) utilized glycodendrimersomes synthetic vesicles formed by the self-assembly of mannose-JDs, whose membrane simulate the surface of a cell, to examine the carbohydrate biological activity [112].

### 7.3. JDs as Nanocarriers

The limitations of free drugs, for example poor water-solubility, toxicity related to a non-selective biodistribution and multi drug resistance, could be prevented using vesicular assemblies as drug delivery systems. As against micelles, vesicles benefit from the ability to encapsulate both hydrophobic and hydrophilic cargos [87].

The suitable self-assembly comportment of amphiphilic JDs has turned into a powerful tactic to obtain nanocarriers [104,140], with strong advantages for drug-delivery applications [60,63,87,111,147].

Both dyes and drugs have been encapsulated in dendrimersomes. Hydrophobic molecules were added to an organic solution of the JD and used for thin film hydration or oil-in-water approaches, resulting in the inclusion of the molecule in the hydrophobic bilayer of DSs. On the other hand, hydrophilic molecules were encapsulated in the hydrophilic cavity of DSs by addition to the water or buffer solution, used forward for hydration in the thin film approach or to the organic phase in the oil-in-water approach. The excess amount was removed via dialysis or size-exclusion chromatography [88].

Thus, the encapsulation of dyes, such as calcein in DSs self-assembled JDs containing bis-MPA-based polyester hydrophilic dendrons G1-G3 and (3,4,5)-trisubstituted Percec-type hydrophobic dendrons [66], carboxyfluorescein in DSs coassembled from JD comprising bis-MPA-based polyester hydrophilic dendrons G2 and (3,5)-disubstituted Percec-type hydrophobic dendrons [74], fluorescein and Nile red in DSs self-assembled from JD bearing hydrophobic photodegradable polyester dendron G3 with o-nitrobenzyl units and hydrophilic (3,4,5) Percec-type dendron G1 with tri(ethylene glycol) monomethyl ether chains [148], were earlier reported. The dye-loaded DSs proved impermeability and stability in time [66], degradation and triggered release upon irradiation with UV light [148], or fluorescence color changes as a function of pH [149].

Until 2018, the most researches regarding the use of JDs as drug nanocarriers refer to the encapsulation of anticancer drugs. Therefore, the inclusion of doxorubicin in DSs self-assembled from either JDs with bis-MPA-based polyester hydrophilic G2 dendrons and (3,5)-disubstituted Percec-type hydrophobic dendrons [66], or JD bearing a hydrophilic poly(amidoamine) dendron and two hydrophobic C18 alkyl chains [150] and Plitidepsin in DSs co-assembled from JD comprising two bis-MPA dendrimers G1-3 of different polarity, the hydrophilic one with free terminal OH groups, and the hydrophobic one functionalized with aliphatic chains derived from stearic acid [151] were reported. The drug-DSs assemblies showed good stability and impermeability [66,151], pH-dependent drug release [66,149], prevention of systemic toxicity [150], or encapsulating ability dependent on the hydrophobic/hydrophilic ratio of dendrons [151].

More recently, DSs were used as extraordinary carriers for various small molecules and macromolecules, being a concrete promise as therapeutic entities for different diseases treatment.

Unique amphiphilic Janus glycopeptide dendrimers, with precise and varied molecular structures containing β-cyclodextrin as core grafted with hydrophilic saccharides as dendrons and hydrophobic peptides as arms, were designed and synthesized by Bi and colleagues (2022). They were self-assembled into different controllable nanostructures such as glycospheres, worm-like micelles, and fibers conditioned by the repeating unit fraction of saccharides and phenylalanine. These nanomaterials were successfully encapsulated with Nile red dye and fluorofenidone, an anti-inflammatory agent [144].

Redox-sensitive DSs, containing disulfide-linked cholesterol-bearing PEGylated dendrimers, were also able to entrap both the lipophilic Nile red and the hydrophilic dye rhodamine. The redox-triggered release of the encapsulated lipophilic dye from the cholesterol-based DSs was assessed using glutathione as a reducing agent [113]. Rhodamine B was also encapsulated in a triazolylferrocenyl JD micelles (Table 2) and released based on the redox response of the Fc moiety [93]. On the other hand, hydrophobic fluorescent probe molecules, *N*-phenyl-1-naphthylamine and coumarin-153, were entrapped in DSs obtained by spontaneous self-assembly of a disulfide-linked octadecyl chain modified PEGylated dendrimer, which showed a redox-responsive disassembly at a glutathione concentration similar to the one of the intracellular media [152].

**Figure 7:**
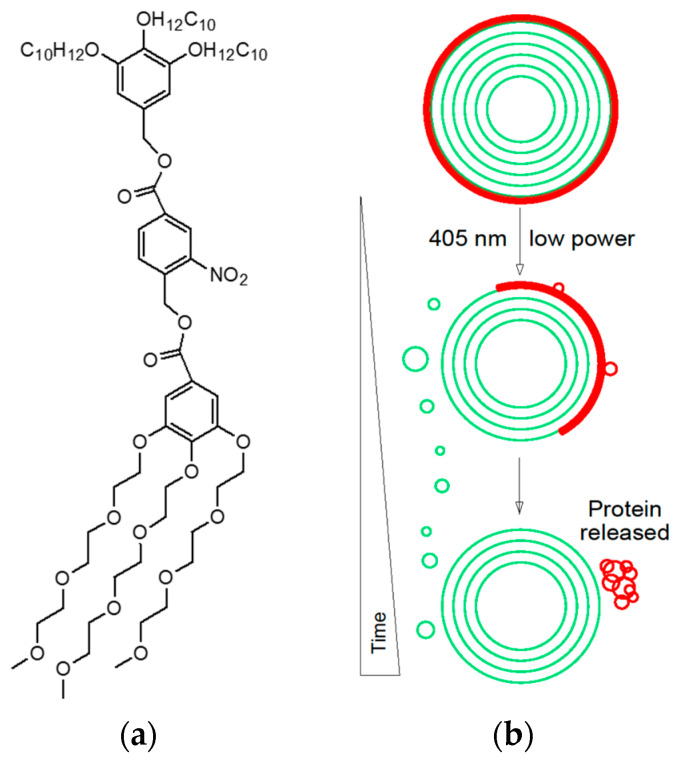
Light-responsive dendrimersome vesicle platform. (**a**) Example of photocleavable Janus dendrimer; (**b**) Representation of light-induced release of attached protein cargo of the outer JD vesicle layer [153].

Because the release of loaded molecules from cell-like nanocarriers can be accomplished by optical modulation of membrane properties, and based on the chemical flexibility of JDs, Li et al. (2020) designed an ideal self-assembling light-responsive dendrimersome vesicle platform, starting from JDs containing ortho-nitrobenzyl photolabile core between the lipophilic and hydrophilic dendrons (Figure 7). Supramolecular structures such as unilamellar, multilamellar, and onion-like vesicles, after milliseconds to seconds of illumination, photocleave, disassemble, and reassemble. If loaded with small molecules (Bodipy, a dye, and doxorubicin, an anticancer drug) or macromolecules (His-tagged red fluorescent protein and *E. coli* dihydrofolate reductase with an *N*-terminal fusion to glutathione-S-transferase and green fluorescence protein), they photocleave and release up to 90% of their cargo, proving the feasibility of such JDs in an optically controlled recruitment and release of encapsulated components [153].

Najafi et al. (2020) designed new JDs containing poly(propylene imine) G1-5 as hydrophobic dendron and PAMAM with NH_2_ end groups as hydrophilic dendron used for increasing drug solubility in water of tetracycline, an antibiotic, and dexamethasone, and an anti-inflammatory agent. The role of hydrophobic dendron was to encapsulate the hydrophobic drug, while the solubility in water was achieved by the hydrophilic one. The improvement of the solubility was dependent on concentration and generation of the used dendrimer [111].

Special attention has been paid in recent years to the use of JDs as carriers for antivirals. Though the existing antiviral agents are efficacious against some viral infection, side effects and acquired resistance can limit their use.

New, broad-spectrum antimicrobial agents are immediately required to efficiently treat some infection diseases [154]. There are some anti-HCV medicines, i.e., camptothecin [147], iopanoic acid, tiratricol [114], acting as bioactive inhibitors of viral NS3 protease, unstable at physiological pH [147] or with no precise activity in inhibition of viral replication in cell-based tests due to cell internalization problems [114]. These issues can be resolved using JDs nanocarriers owing exceptional properties, positive input on drug pharmacokinetics favoring drug bioavailability, drug targeting and cellular internalization [114,147,155,156].

Amphiphilic JDs obtained via combination of 2,2′-bis(hydroxymethyl)propionic acid (bis-MPA) and 2,2′-bis(glyciloxy)propionic acid (bis-GMPA) functionalized with hydrophilic ammonium or stearic acid lipophilic groups (Table 1) were used to encapsulate iopanoic acid and tiratricol antiviral drugs. The best two antiviral combinations in the meaning of cytotoxicity absence in normal cells and inhibitory effects on HCV replication [114] are shown in Table 2. Another anti-HCV drug, camptothecin (CPT), was earlier encapsulated either in JDs based on different generations bis-MPA dendrons terminated in ammonium and stearic acid groups [147], or in bis-GMPA dendrimers having inner amide groups [157]. CPT-nanocarriers proved to be less toxic and the solubility of CPT was increased [147,157].

The importance of JDs in the antiviral fight has become evident in recent years. JDs can act not only as guests for inclusion of some antiviral agents, but also as anchor for antiviral peptide sequences. The last approach can initiate the development of a novel strategy for obtaining therapeutically relevant drugs to fight against many global threatening viral infections. Some examples in this regard can be found in Section 7.4.

Besides, JDs can be used as prodrugs for anti-inflammatory agents. Very recently, a (bis-MPA) Janus-type dendrimer, having multiple naproxen ends in one dendron and multiple ibuprofen ends in the other dendron, was designed and tested for its antiproliferative activity against leukemia and colorectal cancer cells. The antitumoral activity of this ibuprofen-naproxen-Janus conjugate, superior to the one of free drugs and similar to the one of acknowledged anticancer cisplatin, and the lack of toxicity observed for normal cells recommend this kind of conjugates for biomedical application as drug delivery vehicles [158].

The properties of JDs, including unique and specific structures, bioactivity, targeting, and controllable cargo release, could provide a new pattern for the development of smart and structure–controllable nanomaterials with great potential for biological applications, such as target delivery and release of therapeutic and bioactive drugs.

### 7.4. JDs as Protein Recruitment Enhancers

Synthetic polymers [159,160,161,162] and nanoscale supramolecular assemblies like dendrimers [163,164,165] were studied as delivery systems for therapeutic peptides that target intracellular proteins. Despite much research in this area, there are still many challenges, like the difficulty of formulation which influences the physicochemical properties and bioactivity of peptides and the lack of robustness shown by some of these carriers in the delivery of peptides with different size, hydrophobicity, and charge. Hence, the development of a facile and robust approach for delivering peptides that can overcome many extracellular and intracellular impediments remains a challenging task [166].

Decoration of the dendrimersomes surface with proteins in order to enhance the interactions with living cells is a trend, especially since the stability of DSs better tolerates the introduction of functionality compared to liposomes [88].

Choi and co-workers (2019) developed some Janus peptide dendrimers (JPDs) self-assembled into 3D structures analogous to those of globular proteins, where only the dendrimer generation dictates the morphology. The co-assembly of two JPD building blocks gives rare and fascinating architectures, proving the relevance of JPD systems as materials with high avidity for the desired cell types and possibly any target receptors [167].

Remarkable results were obtained by Falanga et al. (2021) who designed a polyamide-based Newkome-type JD scaffold for postsynthesis bioconjugation with peptides derived from the envelope fusion-glycoproteins (gH, gB) of Herpes simplex virus type 1 (HSV-1). The JD structure mimics proteins and ensures biocompatibility and biodegradability of the novel engineered antiviral nanotherapeutics. It is capable of improving the viral inhibition mechanism by multivalent binding, which is concretized in the appearance of irreversible local distortion and loss of infectivity at a much lower peptide concentration. Such JD can be coupled, as needed, with the required peptides for the prevention and treatment of multiple viral infections [168].

Wang et al. (2019) designed and obtained new zwitterionic Janus dendrimers with specific functionalities for effective binding/repelling of targeted proteins. Besides the capability to form protein loaded assemblies via interaction and encapsulation of proteins through hybrid electrostatic and hydrophobic forces, these JD-nanocarriers showed the ability to resist the nonspecific serum protein binding in a biological medium due to the zwitterionic dendrons. JDs containing glycerylphosphorylcholine (GPC) were superior in enhancing the intracellular uptake of protein cancer therapeutics against zwitterionic carboxybetain (CB)-JD and PEGylated JDs. Benefits like biocompatibility, efficient lysosomal escape, controlled in vivo release of insulin and the improved blood sugar control in mice, recommend this JD class for application in therapeutic protein delivery [110].

Without endangering the high lateral mobility for advanced functions and the stability of DSs, the periphery of the DSs can be functionalized with a nitrilotriacetic acid-conjugated Janus dendrimer (NTA-JD), resulting in supramolecular structures with great capacity of binding His-tagged protein cargo [132,169] and a DNA aptamer [132].

Besides NTA-JD, TrisNTA-JD can also be used to co-assemble phospholipids and cholesterol with JDs and block copolymers to give liposomes, dendrimersomes, and polymersomes, respectively. The great protein-binding activity of such hybrid liposomes recommends them as novel resourceful tools for biological reconstitution, synthetic cell biology and nanomedicine [169].

### 7.5. Vectors for Gene Delivery

Gene therapy, a promising therapy for various diseases such as genetic disorders, viral infection and cancers, depends on the adaptable targeting gene delivery systems. Non-viral vectors of gene delivery, composed of biocompatible materials, are less likely to induce an immune response compared to viral vectors, and have to protect the foreign genetic molecule to remain stable within the host cells [170]. Besides non-viral vectors such as liposomes and polymers, which facilitate gene delivery by the formation of lipoplexes and polyplexes, respectively [170], dendrimers that form dendriplexes with nucleic acids stood out due to their characteristic multivalency, precise structure, and significant binding capability together with high efficiency for the internalization into target cells [171].

#### 7.5.1. DNA

One of the most frequently used gene therapy techniques consists of recombinant DNA technology, in which the gene of interest, or a healthy gene is inserted into a vector able to carry therapeutic DNA and to selectively deliver the extrachromosomal material to target cells, without side effects to healthy tissues. The main focus of this technique is the optimization of delivery vectors [172].

In this regard, disulfide-linked cholesterol-bearing PEGylated G3 diaminobutyric-polypropylenimine dendrimers formed DSs which proved the ability to condense DNA instantly, being stable for at least 24 h. These vesicles resulted in an enhanced cellular uptake of DNA, and increased the gene transfection on the PC-3 prostate cancer cells [113]. Further study reports for the first time the prodrug camptothecin-bearing PEGylated polypropylenimine dendrimer, which creates spontaneous, redox-sensitive, 7 days stable cationic DSs, with a supplementary ability to bind via electrostatic interactions to negatively charged phosphodiester groups of DNA, leading to improved gene expression in the prostate cancer cell line. Together, camptothecin and DNA were co-localized in the target cells nuclei [173]. Another example of dendriplexes is the one obtained from self-assembled disulfide-linked octadecyl chain modified PEGylated dendrimer, which has the ability to condense DNA, forming positively charged vesicular-shaped dendriplexes with enhanced cellular uptake of DNA on PC-3 and DU145 prostate cancer cell lines [152].

Based on these facts, it can be stated that the DSs are auspicious gene delivery systems for potential applications in cancer therapies, that can combine DNA and chemotherapeutics for optimized efficiency.

#### 7.5.2. Messenger RNA

Messenger RNA (mRNA) is an emerging class of therapeutic agents used for prophylaxis and treatment of various diseases. Due to the recent triumph of the two highly effective mRNA COVID-19 vaccines, the enormous power of mRNA technology to reform life science and medical research is evident [174]. Issues referring to stability and immunogenicity of mRNA, in vivo distribution and the ability to pass various biological membranes and target selective organs, were widely debated [174,175]. The selective organ targeting strategy was employed for lipid nanoparticles used in mRNA delivery and might cover further nucleic acid therapeutics, providing the approach for a target organ delivery [175]. This key obstacle for clinical applications of nucleic acid therapeutics was addressed recently in the work of Zhang et al. (2021) [72,176]. They designed a synthetic delivery system for mRNA based on one-component multifunctional sequence-defined ionizable amphiphilic Janus dendrimer. Thus, 54 well-defined ionizable amphiphilic JDs were synthesized via an accelerated modular-orthogonal approach. The hydrophilic dendron hold sequence-defined configurations of selected ionizable amines, dimethylamino-acetate/propanoate/butanoate and piperidine/methylpiperazine-butanoate, while the hydrophobic dendron hold linear and branched alkyl radicals of different length. These JDs were used to obtain dendrimersome containing mRNA nanoparticles (mRNA-DNPs). Via a simple injection in acetate buffer, 98% of mRNA was encapsulated in the inner of the DNPs. Several in vitro active mRNA-DNPs, stable at 5 °C for more than 135 days even in serum, exhibited higher transfection efficiency at lower ionizable amine concentration and organ specificity, with the highest luminescence intensity observed in the lung [72]. After some changes in the structure of hydrophilic dendron, like removal of its almost entire hydrophilic part but keeping ionizable amine, and replacement of the interconnecting moiety at the hydrophobic dendron from amide to ester, new supramolecular architectures were obtained. The protonated ionizable amines also acted as binding ligands for mRNA, and modification of the mRNA delivery from lung to spleen and/or liver was achieved. Thus, such simple one-component ionizable amphiphilic Janus dendrimer formulation, easy to transform when needed, and with appropriate size and delivery efficacy, represent promising vectors for mRNA delivery for future biomedical applications [175].

#### 7.5.3. Small Interference RNAs

Small interference RNAs (siRNAs) constitute one of the most important progresses able to regulate gene expression, showing promising therapeutic results for a good number of diseases, particularly in cancer. The advance of effective siRNA delivery systems in nanomedicine is based on the successful siRNA delivery in vivo, so the development of novel systems with exceptional stability, high reproducibility and lower toxicity is mandatory, due to the critical role of delivery systems in the clinical application [177].

siRNA lipid-based nano-formulations are already scaled up [178], but their instability and toxicity limit their clinical value [179]. Besides, cationic polymers, which are stable and biocompatible agents, can only be synthesized with low reproducibility [180]. Thus, using JDs as siRNA delivery systems seems to offer the opportunity of combining conveniently synthesis and high reproducibility with negligible cytotoxicity, excellent biocompatibility and rapid intracellular siRNA release from nanoparticles improving gene silencing efficacy and safety of such gene delivery vectors [107].

On the other hand, JDs have arisen as promising siRNA carriers due to their well-defined structure, appropriate multivalency, and great ability to carry a large cargo loaded within a nanosized volume [88].

Cationic Janus dendrimers merge the multivalency of dendrimer vectors with the self-assembly of lipid vectors, providing robust and versatile nanoassemblies for siRNA delivery [97,98,107,181,182,183].

Du and colleagues developed an original amphiphilic Janus dendrimer comprising a cationic hydrophilic dendrimer with three amino groups, and a hydrophobic dendrimer with two fatty chains bearing disulfide bonds. They self-assembled into a redox-sensitive dendrimersome able to efficiently bind the siRNA by electrostatic interactions, carry the siRNA into cancer cells and release of siRNA by disulfide bond cleavage in the redox environment of tumor cells [107].

Very promising results were obtained by Dong and coworkers, who report the first example of a targeting strategy employed for self-assembling dendrimer-mediated siRNA delivery. Small amphiphilic Janus dendrimer-based delivery system was equipped with the dual targeting warhead RGDK peptide, which successfully directed the specific delivery to PC-3 prostate cancer cells within the tumor, enhanced gene silencing and spared the other cells from toxicity [98].

### 7.6. JDs as MRI Traceable Probes

Nanocarriers like liposomes and polymersomes are well known for their application in magnetic resonance imaging (MRI) [184]. Still, the use of self-assembled Janus dendrimers is related to lower price and easier preparation. Therefore, dendrimersomes containing both MRI contrast agents and chemotherapeutics could be used as theranostic cancer treatments [88].

Beside some low and higher generation JDs, co-assembled into DSs and loaded with fluorescent and magnetic resonance imaging (MRI) contrast agents with good theranostic potential [74,75,185] and in vivo proved safety and stability profiles [186], and those used as surface ligands to obtain biocompatible UCNP/Janus-dendrimers with higher colloidal stability and remarkable feat as vascular markers even at high scanning speeds [76], outstanding results were obtained in case of Janus fluorodendrimers (JFDs) in the field of magnetic resonance imaging.

#### ^19^F-MRI Traceable Probes

The ^19^F magnetic resonance imaging (^19^F MRI) represents a promising tool for the examination of complex physiological and pathological processes, including detection of tumor biomarkers, tracking of functional cells and injury-related biomolecules, and visualization of disease evolution. This technique will bring noteworthy contributions to precision medicine, if its drawbacks such as low sensitivity and limited types of probes can be eliminated [187].

Among dendrimers, the JDs were pioneers in ^19^F MRI field [35]. Thus, small bispherical JDs, exhibiting a single ^19^F NMR line, were obtained using an unelaborate procedure and proved not to be retained in the organs [188], a requirement needed for imaging tracers.

Later on, more elaborated JFDs structures were designed, having a higher number of near completely chemically equivalent fluorine atoms and narrow polydispersity, which co-assembled into dendrimersomes [189].

Recently, Rosati et al. (2022) designed new fluorinated Janus-type dendrimers up to G3 and obtained them using a click chemistry method to link hydrophilic and hydrophobic dendrons. They proved the dependence between the morphology of supramolecular structures and the primary structures of the dendritic building wedges, also mentioning the critical role of the cosolvent. The G1 and G2 FJDs formed spherical nanoparticles in water, with a single and stable ^19^F-NMR peak and good signal-to-noise ratio [77], so the potential of using them as ^19^F-MRI traceable probes must be considered.

Thus, the design of new structures, ^19^F probes, like fluorinated Janus-type dendrimers to reach high fluorine content, appropriate hydrophilicity, and high specificity for the biological markers, constitute a huge potential for medical research and clinical practice.

Although few in vivo studies regarding fluorodendrimers are available, the power of these promising MRI agents should promote them for applications that strongly benefit from molecular quantification [35].

## 8. Conclusions and Future Perspective

The research in the field showed that studying Janus dendrimers is a complex process, starting with suitable design and synthesis, the election of proper methods for characterization both for Janus dendrimers and their combination with active moieties (because only well-defined structures are suitable for obtaining safe and effective nanocarriers), and ending with the demonstration of their applicability. Obtaining JDs is a difficult process requiring elaborated multi-step syntheses, somehow eased by the development of highly chemo-selective methods. However, more research is needed to ensure simpler and cost-effective procedures, that could be implemented for commercial production of JDs. The exploration of possible applications of JDs is still in the early stages, so, fortunately, new and valuable possibilities to exploit JDs will soon be discovered. Thus, more in vivo studies must be implemented that should focus on the stability, pharmacokinetic properties, bioavailability and toxicity of various drug-JDs assemblies. However, this review demonstrates the great potential of amphiphilic Janus dendrimers and their corresponding dendrimersomes for biomedical applications. Their particular features, like controllable size, assembly, density, generation and number of peripheral groups, solubility, presence of internal cavities, stability, and biocompatibility, have opened an egress of countless applications for these amazing molecules in pharmacy, nanotechnology and biomedicines. Amphiphilic Janus dendrimers proved to be effective as stabilizing agents for drug suspensions and silver and gold nanoparticles. They also act as biological membrane mimics, allowing to clarify some cellular processes happening in living cells lending themself to exciting biological applications. Still, their main application is the one of nanocarriers, whether we refer to the encapsulation of small molecules like dyes, drugs and MRI traceable probes, or macromolecules such as proteins and nucleic acids, being a concrete promise as therapeutic entities for the treatment of different diseases. Thus, dendrimersomes act as multifunctional envelopes for various classes of agents, such as anticancer, antiviral, antibiotic, and anti-inflammatory, favoring solubilization, drug bioavailability, drug targeting and cellular internalization, besides controllable cargo release and low toxicity. Then, the exceptional theranostic potential, highlighted for Janus fluorodendrimers and dendrimersomes loaded with chemotherapeutics and MRI contrast agents, recommend them for significant contributions to precision medicine. On the other hand, the ability of forming protein loaded assemblies with high avidity for the desired cells and possibly any target receptors recommend the Janus dendrimers for application in therapeutic protein delivery. Not least, dendrimers which form dendriplexes with nucleic acids are robust and versatile nanoassemblies providing auspicious gene delivery systems for future biomedical applications.

Recent progresses in this field will end in syntheses of Janus dendrimer on a commercial scale, which will provide an advance for medical research and clinical practice.

## Figures and Tables

**Table 2:** Examples of methods implemented for the characterization of some drug-JDs aggregates.

Encapsulated Drug	Hydrophilic/Hydrophobic Dendrons	Methods	Tests	Results	Conclusions
Indomethacin (IN) [106]	Propargyl-modified bis-MPA G3, G4/ Percec-type azide	DSC	Physical interactions Chemical compatibility	- the absence of major discrepancies in the thermal events	- IN remains in crystalline γ-form - unchanged overall crystallinity of the JD-IN
		CAT	Drug wettability	- increasing contact angles	- JD enhance IN particle wetting
		MP-SPR	Solid−liquid interface interactions	- irreversible adsorption of JD	- stabilization of IN particles based on steric hindrance
		DLS	Stabilization of IN suspensions	- IN particle size reduction -	- stabilization of IN particles in the presence of G4 JD
		UV-Vis	Dissolution rate	- dissolution rates of IN comparable to the control	- JD stabilize the IN milling suspensions
Rhodamine B (RhB) [93]	TEG termini /Ferrocenyl (Fc) units	UV-Vis	Drug loading	- presence of characteristic absorption peak of RhB in micelles - drug loading content	- successful loading of RhB into the JD micelles
			Redox- responsive release	- decrease of absorption intensity in time	- controlled release in the presence of FeCl_3_
		DLS	Redox- responsive release	- single size distribution of RhB-loaded micelles, small PDI value - increased average sizes, wide size distribution in presence of FeCl_3_	- RhB could be released easily through the increased gap in micelles
Tiratricol (TRIAC) [114] Iopanoic acid (IA) [114]	(NH^3+^)_8_ [GMPA]/[MPA](C17)_4_ (NH^3+^)_8_ [GMPA]/[MPA](C17)_2_	ITC	JD-drugs affinity	- Ka - ∆G, ∆H, −T·∆S	- interaction driven by entropic contributions
		UV-Vis	Drug loading	- drug loading content	- successful loading
		TEM	Morphology of JD-drugs	- significant changes - no significant shape disruptions for (NH^3+^)_8_[GMPA]-[MPA](C17)_2_/ drugs - wormlike micelles for (NH^3+^)_8_[GMPA]-[MPA](C17)_4_ /IA, large spherical assemblies - long cylindrical micelles for - (NH^3+^)_8_[GMPA]-[MPA](C17)_4_ /TRIAC, lamellar structures	- successful loading - stabilizing role of bigger hydrophilic dendron - significant increase of lipophilic contents, not compensated by the size of the hydrophilic dendron
